# Supplementary figures and images for: Telomere length as a biomarker for fetal fraction prediction in non-invasive prenatal testing
Source: PLoS One. 2025 Jul 11;20(7):e0327714. doi: 10.1371/journal.pone.0327714 (PMC12250228; doi:10.1371/journal.pone.0327714)

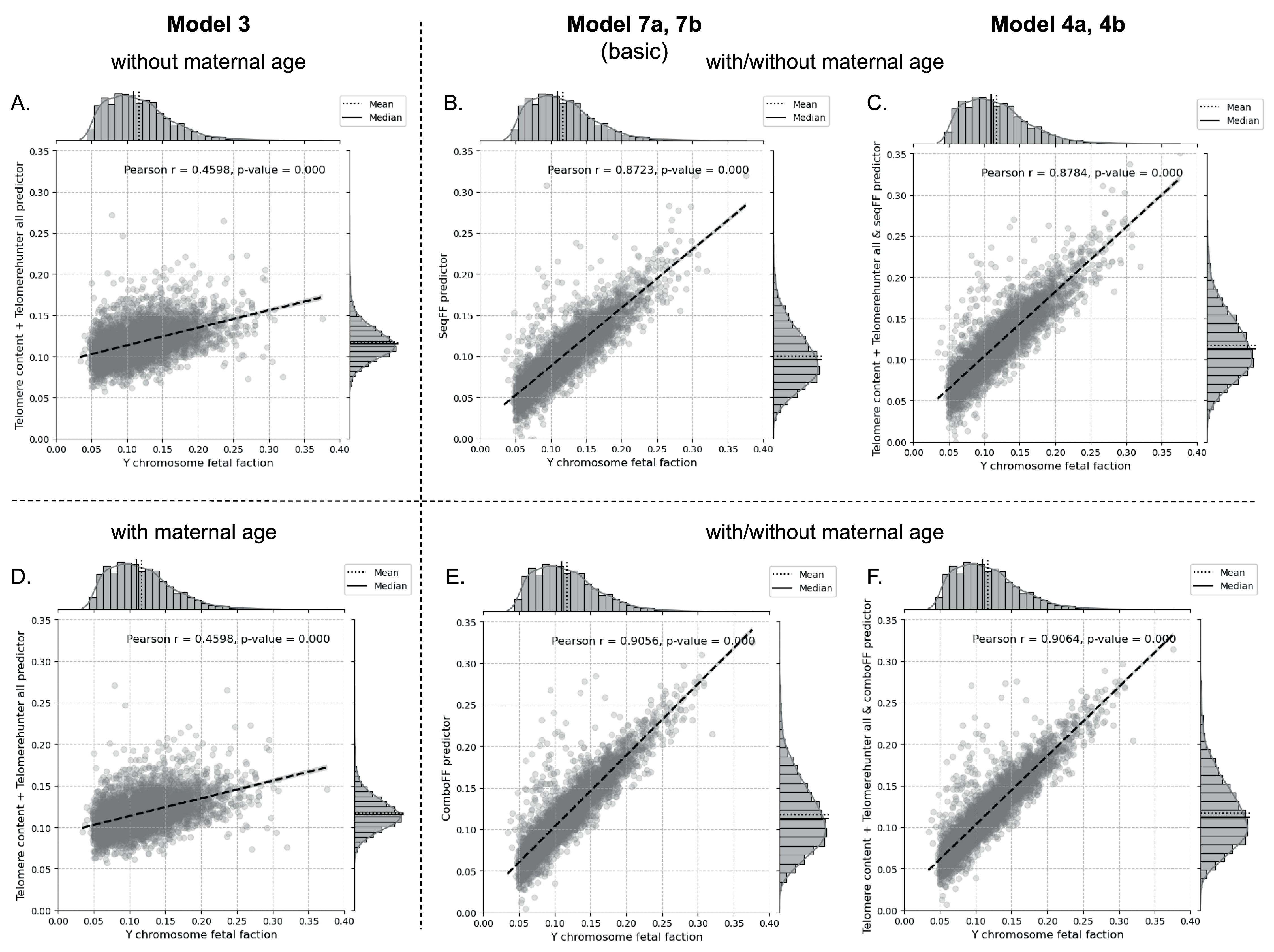

Supplement: S1 Fig — Model 3 demonstrates a better correlation (r = 0.4598) and a slightly lower RMSE of 0.0397, indicating an improvement over the use of telomere content alone. This model was highly significant (p-values = 8.1e-264/7.438e-264). SeqFF with TelomereHunter features (Model 4a) achieved a correlation of r = 0.8784 and an RMSE of 0.0214. In contrast, comboFF with TelomereHunter features (Model 4b) reached an even higher correlation of r = 0.9064 and the lowest RMSE of 0.0189. For further details, refer to Models 7a and 7b in Fig 6. (TIF) [file pone.0327714.s001.tif]

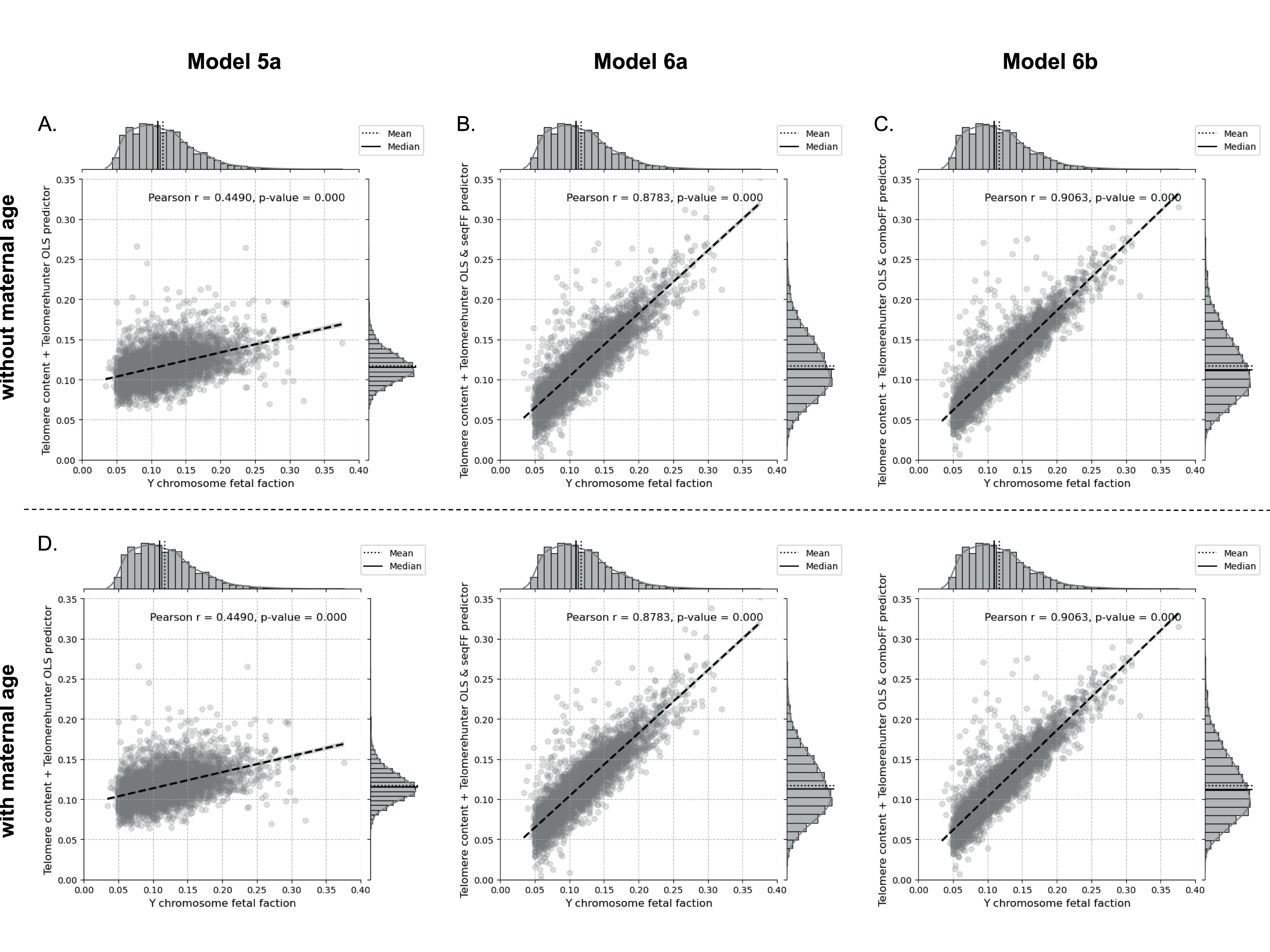

Supplement: S2 Fig — Model 5a, which incorporates OLS-selected features alongside telomere content, yields a moderate correlation (r = 0.4490/0.4490) and an RMSE of approximately 0.0399, indicating that while the inclusion of targeted features provides some improvement, it remains less effective than more comprehensive models. In contrast, Model 6a achieves a high correlation of r = 0.8783/0.8783 and an RMSE of 0.0214, affirming the effectiveness of seqFF with selected features. Model 6b reaches r = 0.9063/0.9063 with an RMSE of 0.0189, marking this combination as one of the top-performing configurations. (TIF) [file pone.0327714.s002.tif]
