# Supplementary material for: Telomere length as a biomarker for fetal fraction prediction in non-invasive prenatal testing
Source: PLoS One. 2025 Jul 11;20(7):e0327714. doi: 10.1371/journal.pone.0327714 (PMC12250228; doi:10.1371/journal.pone.0327714)
Supplement: S1 Table — (DOCX) [file pone.0327714.s003.docx]

#### S1 Table. *TelomereHunter* variables.

| ***TelomereHunter* variables:** | | |
| --- | --- | --- |
| tel_reads,  intratel_reads  total_reads_with_tel_gc  **tel_content**  TTAGGG_norm_by_intratel TCAGGG_norm_by_intratel TGAGGG_norm_by_intratel TTTGGG_norm_by_intratel CTTGGG_norm_by_intratel GTTGGG_norm_by_intratel AGTGGG_norm_by_intratel CTCGGG_norm_by_intratel GGGGGG_norm_by_intratel ATTGGG_norm_by_intratel ACTGGG_norm_by_intratel GGAGGG_norm_by_intratel GGTGGG_norm_by_intratel  GAAGGG_norm_by_intratel | CTAGGG_norm_by_intratel AGAGGG_norm_by_intratel GCTGGG_norm_by_intratel AAAGGG_norm_by_intratel TAAGGG_norm_by_intratel CATGGG_norm_by_intratel CCAGGG_norm_by_intratel CAAGGG_norm_by_intratel AATGGG_norm_by_intratel GTAGGG_norm_by_intratel GGCGGG_norm_by_intratel GATGGG_norm_by_intratel AGCGGG_norm_by_intratel  ACAGGG_norm_by_intratel TGTGGG_norm_by_intratel TCTGGG_norm_by_intratel GCAGGG_norm_by_intratel CGTGGG_norm_by_intratel | CCTGGG_norm_by_intratel ATAGGG_norm_by_intratel ACCGGG_norm_by_intratel GACGGG_norm_by_intratel TATGGG_norm_by_intratel GTCGGG_norm_by_intratel CACGGG_norm_by_intratel TCCGGG_norm_by_intratel CGAGGG_norm_by_intratel CCCGGG_norm_by_intratel TTCGGG_norm_by_intratel GCCGGG_norm_by_intratel AACGGG_norm_by_intratel TGCGGG_norm_by_intratel TACGGG_norm_by_intratel ATCGGG_norm_by_intratel CGCGGG_norm_by_intratel |
