# Supplementary material for: Telomere length as a biomarker for fetal fraction prediction in non-invasive prenatal testing
Source: PLoS One. 2025 Jul 11;20(7):e0327714. doi: 10.1371/journal.pone.0327714 (PMC12250228; doi:10.1371/journal.pone.0327714)
Supplement: S2 Table — 1quantity of input DNA used in the sequencing process, 2concentration of the final prepared sequencing library, 3gestational age refers to the age of the fetus in days, 4total count of reads containing telomeric sequences, 5counts intratelomeric reads or reads with variations in the standard telomeric repeat sequence, 6total count of reads containing telomeric sequences, including GC-content adjustments, 7telomere content in the sample, calculated as the proportion of reads containing telomeric repeats (e.g., TTAGGG sequences) relative to total reads, 8-14normalized count of reads containing the exact TTAGGG/TCAGGG/TGAGGG/TTTGGG/GTTGGG/AGTGGG/AGAGGG repeat sequence divided by the number of intratelomeric reads. 4-14OLS-selected features from TelomereHunter, 1-3others OLS‑selected features. (DOCX) [file pone.0327714.s004.docx]

#### S2 Table. Ordinary least squares (OLS) regression results.

| **Variable** | **Coefficient** | **Std. Error** | **t-value** | **p-value** |
| --- | --- | --- | --- | --- |
| const | 0.1575 | 0.012 | 12.908 | 1.34e-37 |
| input-dna_quantity^1^ | -0.0094 | 0.002 | -4.42 | 1.00e-05 |
| input-final_library_concentration^2^ | -0.0015 | 0 | -11.069 | 3.42e-28 |
| input-gestational_age^3^ | 2.98e-05 | 1.25e-05 | 2.384 | 1.72e-02 |
| tel_reads^4^ | -2.28e-06 | 6.08e-07 | -3.755 | 1.75e-04 |
| intratel_reads^5^ | 4.93e-06 | 1.13e-06 | 4.36 | 1.32e-05 |
| total_reads_with_tel_gc^6^ | 1.68e-08 | 5.46e-09 | 3.076 | 2.11e-03 |
| tel_content^7^ | 2.55e-05 | 5.93e-06 | 4.298 | 1.75e-05 |
| TTAGGG_norm_by_intratel^8^ | 0.1338 | 0.006 | 22.263 | 2.15e-105 |
| TCAGGG_norm_by_intratel^9^ | -0.169 | 0.033 | -5.184 | 2.25e-07 |
| TGAGGG_norm_by_intratel^10^ | -0.2233 | 0.042 | -5.359 | 8.68e-08 |
| TTTGGG_norm_by_intratel^11^ | -0.0462 | 0.013 | -3.524 | 4.29e-04 |
| GTTGGG_norm_by_intratel^12^ | -0.2915 | 0.065 | -4.504 | 6.81e-06 |
| AGTGGG_norm_by_intratel^13^ | -0.3212 | 0.102 | -3.146 | 1.66e-03 |
| AGAGGG_norm_by_intratel^14^ | 0.5348 | 0.193 | 2.774 | 5.56e-03 |

**^1^**quantity of input DNA used in the sequencing process, **^2^**concentration of the final prepared sequencing library, **^3^**gestational age refers to the age of the fetus in days, **^4^**total count of reads containing telomeric sequences, **^5^**counts intratelomeric reads or reads with variations in the standard telomeric repeat sequence, **^6^**total count of reads containing telomeric sequences, including GC-content adjustments, **^7^**telomere content in the sample, calculated as the proportion of reads containing telomeric repeats (e.g., TTAGGG sequences) relative to total reads, **^8-14^**normalized count of reads containing the exact TTAGGG/TCAGGG/TGAGGG/TTTGGG/GTTGGG/AGTGGG/AGAGGG repeat sequence divided by the number of intratelomeric reads. **^4-14^**OLS-selected features from *TelomereHunter*, **^1-3^**others OLS‑selected features.
