# Supplementary material for: Telomere length as a biomarker for fetal fraction prediction in non-invasive prenatal testing
Source: PLoS One. 2025 Jul 11;20(7):e0327714. doi: 10.1371/journal.pone.0327714 (PMC12250228; doi:10.1371/journal.pone.0327714)
Supplement: Attachment — Submitted filename: Rebuttal letter.docx [file pone.0327714.s007.docx]

Dear Editorial Office,

We thank the reviewers for their generous comments on the manuscript. We have revised the manuscript to address their concerns.

## **Reviewer #1:**

The authors explore the hypothesis that telomere length could serve as a novel biomarker for predicting fetal fraction (FF) in non-invasive prenatal testing (NIPT). While the integration of telomere‑related metrics into applied models (e.g., seqFF and comboFF) provides modest improvements, the overall impact appears limited compared to established genetic-based methodologies. Nevertheless, this research offers valuable insights into potential supplementary approaches for FF estimation. I would like to share some comments and suggestions to enhance the manuscript's clarity and rigor.

**Major Comments:**

- The correlation coefficients reported (e.g., telomere content alone vs. best-performing model) suggest that telomere metrics provide only incremental improvements when integrated with conventional tools. Consider elaborating on the practical implications of these modest gains (e.g., Would the improved prediction accuracy significantly impact clinical outcomes or test reliability?).

***Authors:*** *While the demonstrated slight improvement in prediction accuracy may not significantly influence clinical outcomes, every enhancement in methodologies contributes incrementally to achieving better and more precise results, thereby potentially reducing false clinical outcomes.*

- The manuscript would benefit from a deeper insights of the biological variability of telomere length across populations (e.g., potential influences of maternal health, genetic background, or environmental factors on telomere dynamics). Furthermore, a discussion is necessary on whether the observed telomere-derived signals are consistent across diverse maternal-fetal conditions (e.g., healthy fetuses vs affected).
- Although the models incorporate telomere metrics and maternal age, the manuscript does not sufficiently address potential biases or limitations (e.g., the exclusion of pregnancies with female fetuses; technical variability in telomere measurement; the impact of maternal BMI or other confounding factors). Including a limitations section to discuss these points would strengthen the study.

***Authors:*** *We have expanded the „Introduction“ section to provide deeper insights into telomere dynamics, including their maintenance mechanisms in germ and stem cells via telomerase. Additionally, we have incorporated a dedicated „Limitations“ section discussing telomere variability due to factors such as sex differences, racial and ethnic diversity, and health conditions like obesity, diabetes, and cardiovascular disease and also genetic background, and environmental influences.*

*This study is among the few investigating fetal fraction prediction using telomere content. To ensure accuracy, it focuses on pregnancies with healthy male fetuses, utilizing the Y-chromosome as a reference for fetal fraction estimation and establishing a baseline for telomere-based predictions. While this approach helps reduce certain confounding factors, it may limit the applicability of the findings to pregnancies involving female fetuses or those affected by chromosomal abnormalities or other conditions. Future research should explore these factors to improve the generalizability of telomere‑based fetal fraction prediction.*

*While this approach helps minimize some confounding factors, it may limit the generalizability of our findings to pregnancies with female fetuses or those affected by chromosomal abnormalities. This limitation has now been explicitly addressed in the „Limitation“ section.*

*We also clarify that maternal body mass index (BMI) was included as a parameter in the comboFF model, addressing a key confounding factor in fetal fraction estimation. This is now explicitly stated in the revised manuscript.*

- Indicate as supporting data will be shared to improve transparency and reproducibility.

***Authors:*** *Supporting data will be available in the form of a spreadsheet (supporting_data.xlsx).*

**Minor suggestions:**

- Line 23: placental trophoblast is the main but not sole source of cffDNA. A lesser source is apoptosis of erythroblasts in the fetal circulation, which generates cfDNA that can cross the placenta and enter the maternal circulation (PMID: 11113889; 12200465).

***Authors:*** *Added the word "mainly".*

- Ensure consistent use of terms like “telomere content,” “TelomereHunter features,” and “fetal fraction prediction models” throughout the manuscript.

***Authors:*** *In some cases the word “length” has been replaced by word “content”, the words “variables” and “attributes” by word “features”.*

- The Introduction is effective but could provide more context on the limitations of current FF estimation methods, highlight the potential gaps that telomere metrics aim to fill.

***Authors:*** *The „Introduction“ section has been updated to include limitations of current FF estimation methods with additional information on the potential role of telomeres in FF estimation.*

## **Reviewer #2:**

The accuracy of methods for quantifying fetal DNA fraction in maternal plasma samples during pregnancy is maximized by two main factors, fetal sex (M) and sequencing coverage (>10M reads). In the absence of these requirements, further criteria for defining FF require robust statistical processing dependent on data variability. The search for new models must overcome this last problem. This is not the case for telomere sequences which, although decreasing with age, do not easily distinguish placenta from maternal lymphocytes due to the innumerable inter and intra-individual variables. It is therefore not surprising that the authors did not draw favorable conclusions in their work, nor does it appear useful to share such data.

***Authors:*** *Thank you for your opinion. Although the results of this study did not lead to favorable conclusions, it is important to recognize the value of negative findings. Even these results contribute to our broader understanding and help guide the search for new approaches in this field. For this reason, sharing such data remains crucial, as it enriches scientific discourse and advances progress.*

## **Reviewer #3:**

This study investigates the potential utility of telomere content as an additional biomarker to enhance the estimation of fetal fraction (FF) in non-invasive prenatal testing. The manuscript is clearly written, methodologically sound, and well-structured. The results suggest that telomere length may serve as a “complementary tool” for refining established FF prediction methods. Although the inclusion of telomere-related features yielded only modest improvements in FF prediction accuracy, the study provides valuable insights and highlights the importance of integrating multiple biomarkers to advance FF estimation models.

***Authors:*** *Thank you for your feedback. We appreciate your recognition of the clarity and methodological rigor of our study. We agree that while the inclusion of telomere-related features led to only modest improvements in fetal fraction (FF) prediction, it underscores the potential value of integrating multiple biomarkers into existing FF estimation models. We believe that the combination of various approaches will be key to refining prediction accuracy, and this study serves as a step in that direction.*

------------------------------------------------------------------------------------------------------------------------------------

***Authors:*** *During the process of addressing the reviewers' comments, we identified a minor error in the calculations (Table 1, S2 Fig, and the corresponding text in the „Results“ section). We have now corrected this to ensure accuracy.*

We believe that the manuscript is now suitable for publication in PlosOne.

Zuzana Holesova

On behalf of all authors.
